# Degraded Impairment of Emotion Recognition in Parkinson's Disease Extends from Negative to Positive Emotions

**DOI:** 10.1155/2016/9287092

**Published:** 2016-07-31

**Authors:** Chia-Yao Lin, Yi-Min Tien, Jong-Tsun Huang, Chon-Haw Tsai, Li-Chuan Hsu

**Affiliations:** ^1^Graduate Institute of Neural and Cognitive Sciences, China Medical University, No. 91 Hsueh-Shih Road, Taichung, 40402, Taiwan; ^2^Department of Psychology, Chung Shan Medical University, Taichung, Taiwan; ^3^Neuroscience Laboratory, Department of Neurology, China Medical University Hospital, Taichung, Taiwan; ^4^School of Medicine, China Medical University, Taichung, Taiwan

## Abstract

Because of dopaminergic neurodegeneration, patients with Parkinson's disease (PD) show impairment in the recognition of negative facial expressions. In the present study, we aimed to determine whether PD patients with more advanced motor problems would show a much greater deficit in recognition of emotional facial expressions than a control group and whether impairment of emotion recognition would extend to positive emotions. Twenty-nine PD patients and 29 age-matched healthy controls were recruited. Participants were asked to discriminate emotions in Experiment  1 and identify gender in Experiment  2. In Experiment  1, PD patients demonstrated a recognition deficit for negative (sadness and anger) and positive faces. Further analysis showed that only PD patients with high motor dysfunction performed poorly in recognition of happy faces. In Experiment  2, PD patients showed an intact ability for gender identification, and the results eliminated possible abilities in the functions measured in Experiment  2 as alternative explanations for the results of Experiment  1. We concluded that patients' ability to recognize emotions deteriorated as the disease progressed. Recognition of negative emotions was impaired first, and then the impairment extended to positive emotions.

## 1. Introduction

Parkinson's disease (PD) is a neurodegenerative disorder characterized by a loss of dopaminergic neurons in the substantia nigra par compacta. Dysfunction of the nigrostriatal dopaminergic pathway impairs the function of basal ganglia-thalamocortical circuits, including those in the motor and prefrontal cortex [1]. Patients with PD typically show not only motor symptoms but also cognitive deficits [2–4] and a deficit in the processing of emotional stimuli [5, 6]. Clark et al. (2008) found that PD patients exhibited selective impairment in the recognition of angry and surprised facial expressions [7]. Baggio et al. (2012) reported that PD patients showed reduced recognition of sad, angry, and disgusted facial expressions [8]. A meta-analysis indicates that patients with PD were more impaired in recognizing negative emotion than relatively positive emotion [6].

Previous studies have revealed deficits in PD patients' recognition of facial expressions reflecting only negative emotions such as fear, sadness, anger, and disgust [6–9]. This problem is related to disturbances in the limbic loop, one of the basal ganglia-thalamocortical circuits, and specifically in the connections of the basal ganglia to the orbitofrontal cortex (OFC) and the anterior cingulate cortex (ACC). The limbic loop plays an important role in emotional and motivational processes [10, 11]. Neuroimaging studies have revealed increased activation of OFC and ACC when participants attempt to recognize facial expressions of emotion [12, 13]. Patients with OFC and/or ACC lesions have shown impaired identification of emotional facial expressions [14]. OFC and ACC were found to be active in the processing of facial expressions of negative emotions such as fear and anger [12, 15, 16]. In addition, ACC has been associated with the processing of happiness [13, 17]. Moreover, reduced ability to recognize both negative and positive emotional signals has been demonstrated in patients with Huntington's disease, implying damage to the basal ganglia [18]. Because of the demonstrated dysfunction of the basal ganglia-thalamocortical circuits, we expected to find that PD patients' ability to recognize not only negative facial expressions, but also positive ones, would be impaired.

Why have past studies not exposed deficits in PD patients' recognition of positive emotions? We suggest two reasons. First, the range of PD symptom severity has been shown to be limited to patients in Hoehn and Yahr's stages II and III [19] or with a score range of 8–30 on the motor section of the Unified Parkinson's Disease Rating Scale (UPDRS-III) [20] in past studies. Because of dopaminergic neurodegeneration, PD patients' motor and cognitive functions deteriorate with time. Dopamine levels have been found to be correlated with patients' performance of recognition of emotional faces [21, 22]. Yip et al. (2003) found that, compared to unilateral PD patients, bilateral patients showed greater deficits on the emotion recognition task regardless of the stimulus modality [23]. Alonso-Recio et al. (2014) found that patients with high disease severity performed worse than healthy controls on a test of working memory that involved recognition of emotional facial expressions, but no such effect was found in patients with low disease severity [24]. This suggests that as the disease progresses, it is very possible that PD patients with advanced motor problems have much greater impairment in emotion processing than PD patients with mild motor problems [21–24]. However, the disease severity of PD patients recruited for past studies was not seriously enough impaired to disrupt the recognition of happy faces (see Table 1) [7–9, 21, 22, 25–30]. The second reason has to do with the fact that happiness is the simplest facial expression [31], and it boasts the added advantage of having the most distinctive configuration of all the basic emotional expressions, as illustrated by the pop-up smile [32–34]. Thus, recognition of happy face might be difficult to disable completely as the disease progresses in PD patients. Therefore, we hypothesized that only when patients are in the more advanced stages of PD will they show impairment in the recognition of positive emotions.

PD patients require various cognitive abilities to recognize facial expressions, but they display a wide spectrum of nonmotor problems, for example, in visuospatial function [35, 36], working memory [37–40], decision making [41, 42], and categorization [43]. Without using a suitable control task, it would be difficult to determine whether PD patients' poor performance on our emotional task in Experiment  1 was due to an inability to discriminate emotions or to deficits in the many relevant cognitive abilities. Therefore, in Experiment  2 we employed a control task, the Fast Gender Identification Task (FGIT), which has task requirements comparable to the Fast Emotion Discrimination Task (FEDT). We adopted the same stimuli for the two tasks, the only difference being that one required the processing of emotion and the other did not.

To test our hypothesis, we used motor symptoms, the most relevant observable consequence of neural degeneration, to evaluate disease severity in PD patients. We recruited PD participants with a wide range of symptom severity (H&Y 1–5) for two experiments. In Experiment  1, the performance of PD patients was compared with that of age-matched healthy controls on the FEDT. In Experiment  2, we used the FGIT as a control task in determining whether the PD patients' impairment in recognizing facial expressions was due to deficits in their processing of emotion.

## 2. Experiment  1

### 2.1. Participants

Twenty-nine idiopathic Parkinson's patients not diagnosed as having depression or dementia were recruited from the China Medical University Hospital. Informed consent was given by each participant prior to the study. All participants met the clinical criteria of the United Kingdom Parkinson's Disease Society Brain Bank [51]. All were examined after they withheld their Parkinsonian medications overnight (off-stage). The severity of clinical motor symptoms of these patients was assessed by Hoehn and Yahr's scale (range from 1 to 5) [19] and the motor section of the Unified Parkinson's Disease Rating Scale (UPDRS-III) [20]. General cognitive function was rated by the Mini Mental State Examination (MMSE) [44]. Depression levels were tested by the Beck Depression Inventory II (BDI-II) [52]. Twenty-nine age-matched healthy controls (HC), 11 males and 18 females, with no history of neurological or psychiatric illness were also recruited. MMSE scores of all control participants were in the normal range (see Table 2). The mean BDI-II score was significantly higher for the PD patients than for the HC group, *t*(32) = 5.19, *p* < 0.01, and Cohen's *d* = 1.36. Mean age and MMSE scores did not differ significantly between the PD and HC groups.

### 2.2. Fast Emotion Discrimination Task

The task stimuli were presented on an IBM-compatible personal computer with a 17-inch calibrated View Sonic color monitor, using Presentation v0.70 software (Neural Behaviour Systems Corporation, 2003). The fixation point was a small white cross subtending a visual angle of 0.30° × 0.30°. Each picture displayed a face, size 11.2° × 16.8°, superimposed on a grey background. The pictures, taken from Ekman and Friesen (1976) [53], were of 10 individuals (5 male, 5 female), each of whom displayed four distinct facial expressions: one positive (happiness) and three negative (sadness, fear, and anger). Each of the 10 happy faces was presented three times and each of the 30 negative faces was presented one time, for a total of 60 presentations. The order of presentation was randomized.

Participants sat in a dimly lit room with the chin on a chinrest 65 cm from the screen. A white cross accompanied by a short 1000 Hz warning tone was presented for 300 ms. The target face was then presented in the center of the screen on a grey background for 300 ms. Participants were asked to discriminate the valence of the target face, positive or negative, by pressing the “B” or “M” key on the computer keyboard. The order of key designations was counterbalanced across participants. Accuracy and response times in identifying the emotional expressions were automatically recorded by the computer.

### 2.3. Statistical Analysis

Because of PD patients' motor problems, previous studies have usually focused on the analysis of accuracy data. However, the reaction time measures in our Fast Emotion Discrimination Task also provide important information, especially concerning the different stages of motor severity in PD patients. Patients in an advanced stage of the disease take longer than those in an early stage to respond to presented emotional faces. To assess response time (RT) and accuracy (ACY) at the same time, the RT and ACY scores were combined to form a single dependent variable, Efficiency, which appropriately weights RT and ACY [54–56]. Specifically, the Efficiency scores were calculated as the proportion of a given participant's mean ACY divided by that participant's mean RT across all responses in a given experimental condition. Although none of the patients had been diagnosed with depression, the mean BDI-II score of the PD group was higher than that of the HC group. To exclude BDI-II scores as a possible confounding factor, we included them as the covariate in an ANCOVA. Then we used post hoc* t*-tests. Effect size estimates were eta-squared (*η* ^2^) with the ANCOVA and biased-corrected Cohen's *d* with the* t*-tests.

### 2.4. Results

#### 2.4.1. Efficiency Scores of the PD Patients and HC Group

**Figure 1:**
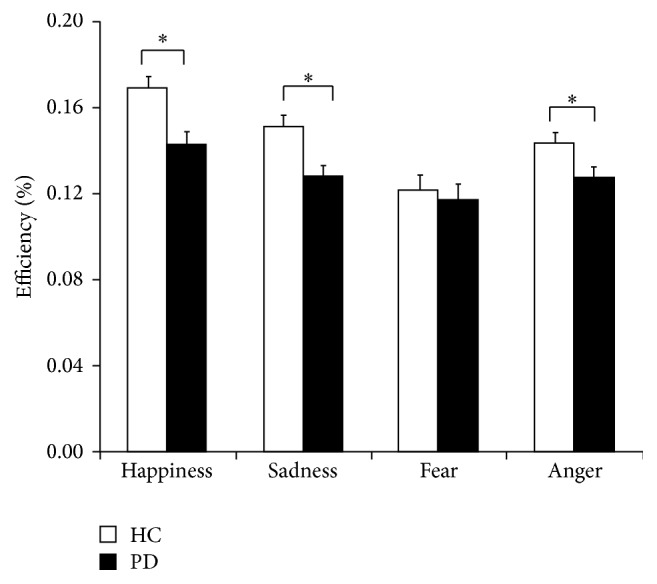
Mean Efficiency scores of the healthy controls (HC) and PD patients (PD) on the Fast Emotion Discrimination Task. Asterisks (*∗*) indicate statistical significance at *p* < 0.05.

Efficiency was assessed first by a 2 × 4 ANCOVA with group (HC, PD) and facial expression (happiness, sadness, fear, and anger) as the independent variables and BDI scores as the covariate. There were significant main effects for facial expression, *F*(3,165) = 15.19, *p* < 0.01, and *η* ^2^ = 0.216, and BDI-II scores, *F*(1,55) = 6.71, *p* < 0.05, and *η* ^2^ = 0.109, and a significant interaction between facial expression and group, *F*(3,165) = 3.15, *p* < 0.05, and *η* ^2^ = 0.054. There was no significant main effect for group and no significant interaction between facial expression and BDI-II scores (*p*s > 0.10). Post hoc analyses show that the PD group performed worse than the HC group on happiness, *t*(56) = 3.11, *p* < 0.01, and Cohen's *d* = −0.82, sadness, *t*(56) = 3.24, *p* < 0.01, and Cohen's *d* = −0.85, and anger, *t*(56) = 2.17, *p* < 0.05, and Cohen's *d* = −0.57. Participants in both groups performed significantly worse on fear than on happiness, sadness, and anger, respectively (*p*s < 0.05). These results indicate that the PD patients were impaired in the recognition of both positive (happy) and negative (sad and angry) facial expressions, and depression was not the main factor affecting patients' emotion recognition. The Efficiency results are illustrated in Figure 1.

#### 2.4.2. Disease Progression and Emotion Recognition

To assess the relationship between disease progression and emotional face recognition in PD patients, we separated the PD patients into two subgroups based on their UPDRS-III scores. The 14 patients (11 male, 3 female) with scores less than the median of 35 were assigned to the low motor dysfunction (LMD) group; the other 15 patients (8 male, 7 female) with scores equal to or greater than 35 were assigned to the high motor dysfunction (HMD) group. As shown in Table 2, mean scores were significantly higher in the HMD group than in the LMD group only on UPDRS-III, *t*(27) = 5.51, *p* < 0.01, and Cohen's *d* = 2.06, and Hoehn and Yahr's scale, *t*(27) = 5.16, *p* < 0.01, and Cohen's *d* = 1.94. Mean age, MMSE scores, and BDI-II scores did not differ significantly between the two PD subgroups (all* p'*s >0.10).

To compare the HC, LMD, and HMD groups, we performed three independent one-way ANOVAs with age, MMSE scores, and BDI-II scores as dependent variables. The three groups did not differ significantly on age or MMSE scores (*p* > 0.1), but they did differ significantly on BDI-II scores, *F*(2,55) = 16.12, *p* < 0.01, and *η* ^2^ = 0.37. Post hoc analyses show that the mean BDI-II score was higher for the LMD group than for the HC group, *t*(14) = 3.16, *p* < 0.01, and Cohen's *d* = 1.16, and higher for the HMD group than for the HC group, *t*(15) = 4.37, *p* < 0.01, and Cohen's *d* = 1.57.

**Figure 2:**
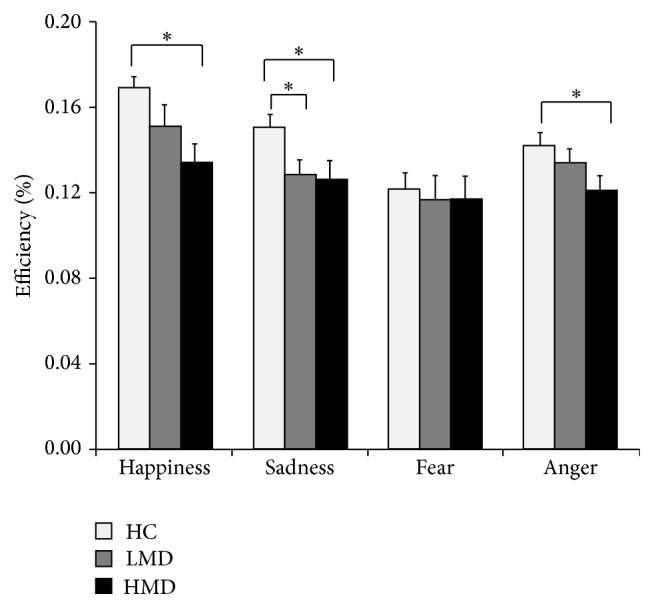
Mean Efficiency scores of the three groups on the Fast Emotion Discrimination Task. HC: healthy controls; LMD: PD patients with low motor dysfunction; HMD: PD patients with high motor dysfunction. Asterisks (*∗*) indicate statistical significance at *p* < 0.05.

Efficiency of the three groups was assessed first by a 3 × 4 ANCOVA with group (HC, LMD, and HMD) and facial expression (happiness, sadness, fear, and anger) as the independent variables and BDI-II scores as the covariate. There were significant main effects for facial expression, *F*(3,162) = 8.69, *p* < 0.01, and *η* ^2^ = 0.139, and BDI-II scores, *F*(1,54) = 5.90, *p* < 0.05, and *η* ^2^ = 0.098, as well as a significant interaction between facial expression and group, *F*(6,162) = 2.49, *p* < 0.05, and *η* ^2^ = 0.084. There was no significant main effect for group and no significant interaction between facial expression and BDI-II scores (both* p'*s >0.10). All participants performed significantly worse on fear than on happiness, sadness, and anger, respectively (*p'*s <0.05). Next, we compared the LMD and HMD groups separately with the HC group by* t*-tests on the recognition of four kinds of facial expression. The analyses revealed that the LMD group performed significantly worse than the HC group only on sadness, *t*(41) = 2.67, *p* < 0.05, and Cohen's *d* = −0.87. The HMD group performed significantly worse than the HC group on happiness, *t*(42) = 3.61, *p* < 0.01, and Cohen's *d* = −1.15, sadness, *t*(42) = 2.66, *p* < 0.05, and Cohen's *d* = −0.84, and anger, *t*(42) = 2.48, *p* < 0.05, and Cohen's *d* = −0.79. These results indicate that the LMD group was deficient only in the recognition of negative facial expressions (sadness), but the HMD group was impaired in the recognition of both positive (happy) and negative (sad and angry) facial expressions. The results further suggest that as the disease advances, PD patients' ability to correctly identify facially expressed emotions declines and the impairment extends to positive emotions. The Efficiency results are illustrated in Figure 2.

## 3. Experiment  2

In Experiment  1, we found that PD patients had deficits in the processing of facial expressions. To confirm whether PD participants' poor performance on tasks of emotion discrimination is due to a disruption of emotion processing, we conducted a nonemotional discrimination task—the Fast Gender Identification Task—as a control task in Experiment  2; we used the same stimuli as in Experiment  1 but asked participants to make gender identifications.

### 3.1. Participants

Three months after completion of Experiment  1, we invited 19 idiopathic Parkinson's patients (PD group) from the same sample to join in Experiment  2. We also recruited 15 new age-matched healthy controls (HC group)—6 males and 9 females. We again separated the PD patients into two subgroups cut at the median score (35) on the UPDRS-III. The 7 PD patients (5 male, 2 female) with scores less than 35 were assigned to the low motor dysfunction (LMD) group, and the 12 PD patients (6 male, 6 female) with scores equal to or greater than 35 were assigned to the high motor dysfunction (HMD) group. The mean UPDRS-III score was higher for the HMD group than for the LMD group, *t*(17) = 4.20, *p* < 0.01, and Cohen's *d* = 2.0. Measured characteristics of all the participants are shown in Table 3. To compare the LMD, HMD, and HC groups, we performed three independent one-way ANOVAs with age, MMSE scores, and BDI-II scores as dependent variables. Only the BDI-II scores differed significantly across the three groups, *F*(2,33) = 3.72, *p* < 0.05, and *η* ^2^ = 0.19. The only significant post hoc effect is that BDI-II scores were higher for the HMD group than for the HC group, *t*(15) = 2.35, *p* < 0.05, and Cohen's *d* = 0.98.

### 3.2. Fast Gender Identification Task

The stimuli and test procedures for the Fast Gender Identification Task were the same as for the Fast Emotion Discrimination Task used in Experiment  1. Participants were asked to identify the gender of the target face by pressing the “B” or “M” key on the computer keyboard. The order of key designations was counterbalanced.

### 3.3. Results and Discussion

Efficiency scores, defined as in Experiment  1, were assessed by a 3 × 2 ANCOVA with group (HC, LMD, and HMD) and gender (male, female) of target face as independent variables and BDI-II scores as the covariate. There were no significant main effects for group, gender, or BDI-II scores, nor were the interactions significant (all* p'*s >0.10). This means that the PD patients could identify gender easily. Thus, patients' difficulty in recognizing facial expressions in Experiment  1 was not caused by any function measured in Experiment  2.

## 4. General Discussion

We aimed to determine the impact of Parkinson's disease progression on patients' ability to recognize emotions. In Experiment  1, we examined the performance of PD patients representing a broad range of motor dysfunction levels. As in previous studies [21, 22, 25] all our PD patients demonstrated a recognition deficit for negative faces (sadness and anger), but further analyses demonstrated that only the HMD patients performed poorly in recognition of happy faces. These analyses collectively demonstrate a positive relationship between disease progression and impairment in the recognition of facial expressions. To our knowledge, this study is the first to demonstrate deficits in the recognition of positive facial emotions in PD patients. As the disease progresses, recognition of negative emotions is impaired first and then the impairment extends to the recognition of positive ones.

Wieser et al. (2006) found that PD patients rated negative pictures as less arousing than did healthy controls, but they had no such problem in accurately rating positive and neutral pictures [57]. Kesler-West et al. (2001) found that normal people had a lower threshold for making a subjective emotional response when they saw a face expressing happiness than when they saw a face expressing a negative emotion [58]. Because happy expressions have a salient and unique facial feature, namely, a smile [59, 60], they are less ambiguous than negative expressions, the reason being that the latter share many overlapping features with one another. The distinctiveness of facial expression features such as the smile facilitates accurate response selection and decision making when one is asked to recognize a happy face [33]. Hence, happiness should be the easiest of the basic emotions to recognize. This means that PD patients can maintain their ability to correctly recognize happy faces until the disease enters its late stage.

To determine whether the poor performance of the PD patients on the Fast Emotion Discrimination Task (FEDT) in Experiment  1 was due to deficiencies in emotion processing per se or to a decline in task-related cognitive functions, in Experiment  2 we gave PD patients the Fast Gender Identification Task (FGIT). The stimuli were the same and the procedures similar in the FEDT and the FGIT. The only difference between the two tasks was whether or not they required the processing of emotion. We found that the PD patients could identify gender easily. Our results in Experiment  2 are consistent with previous studies in that our PD patients could correctly identify nonemotional characteristics such as gender and identity from faces [25]. Thus, the results eliminated deficiencies in abilities such as basic visual spatial ability, decision making, or categorization as alternative explanations for the results of Experiment  1. The PD patients' difficulty in recognizing facial expressions was caused by deficiencies in the processing of emotion.

The processing of happy expressions has been related to activation of the anterior and posterior cingulated gyrus, medial frontal cortex, and orbitofrontal cortex [61, 62]. Evidence from patients with Huntington's disease shows that damage to the basal ganglia impairs the recognition of both negative and positive emotional signals [18]. These studies are consistent with ours in showing that basal ganglia-thalamocortical circuits are involved in the processing of emotions for the recognition not only of negative emotions but also of positive ones.

We found that our PD patients had difficulty in recognizing angry and sad faces, a result consistent with previous studies [21, 22, 25]. The mesolimbic dopaminergic pathway, which includes the ventral striatum and amygdala, evidently plays an important role in the processing of anger and sadness. Imaging studies suggest that the amygdala is responsible for the recognition of sadness [12] and that the ventral striatum is responsible for the recognition of angry faces [63]. Lawrence et al. (2002) found that dopaminergic antagonism selectively disrupted the recognition of facial signals of anger in healthy males [64]. On the other hand, PD patients receiving dopaminergic medication improved more than unmedicated PD patients in the ability to recognize sad faces [21]. PD patients' deficit in the recognition of negative emotions may be related to faulty communication between the amygdala and prefrontal cortex, due to low levels of dopamine.

However, the performance of our PD patients in recognition of fearful faces was not significantly different from that of healthy controls. The participants in both groups performed worse on faces expressing fear than on faces expressing other emotions. Our results are consistent with previous studies in which recognition of a fearful face was found to be more difficult than recognition of a sad, angry, disgusted, or happy face for both PD patients and healthy controls [26]. Rapcsak et al. (2000) attributed the difficulty patients with focal brain damage had in recognizing fearful faces not to the patients' disease, but to difficulty in recognizing fear per se, as was also the case for normal participants [65]. Therefore, it is possible that our failure to find a difference in fear recognition between our patients and healthy controls was due to a floor effect.

The possibility that some of our PD patients were clinically depressed is a limitation of this study, although none were given this diagnosis. Previous studies suggest that depression modulates the processing of emotional information [66]. However, our patients' Efficiency scores did not significantly interact with their BDI-II scores. Previous studies of PD patients have also failed to demonstrate a correlation between accuracy in facial emotion recognition and depression questionnaire scores [22, 25]. Therefore, depression might not be a critical factor in explaining deficits in the processing of facial expressions in PD patients.

The present study was intended to show that the progression of Parkinson's disease affects patients' recognition of facial expressions. Using a cross-sectional design with patients recruited at different stages of the disease progression, we found that more advanced PD patients showed a greater deficit in facial emotion recognition than less advanced PD patients. Future researchers are encouraged to employ longitudinal designs and obtain convergent evidence to track emotion recognition, along with the progression of motor dysfunction. Then we will see a more complete picture of the relationship between Parkinson's disease progression and emotion recognition.

In summary, our study goes beyond earlier research by demonstrating for the first time a relationship between the severity of motor symptoms and impairment of emotion recognition in PD patients, thus highlighting the importance of the effects of PD progression on emotion processing. Further, our PD patients showed processing deficits not only for negative emotions but also for positive emotions as the disease progressed. We conclude that problems of facial expression recognition follow in the wake of neuron degeneration in the dopaminergic system of PD patients.

## Figures and Tables

**Table 1:** Impaired recognition of facial emotion expressions in PD patients.

Author (year)	Patient sample size	Cognitive performance	Mean severity of PD		Impaired emotions
			H&Y	UPDRS-III	
Assogna et al. (2010) [30]	70	MMSE: 27.9		20.1	Disgust

Baggio et al. (2012) [8]	39	MMSE: 28.7		16.5	Sadness, anger, disgust

Clark et al. (2008) [7]	20	DRS-2: 142.6 MMSE: 28.7	2-3 (range)		Anger, surprise

Dujardin et al. (2004) [25]	18	MMSE: >27		17.58	Sadness, surprise

Hipp et al. (2014) [28]	28	MMSE: 28.8 CDR: 0 FAB: 15.46		8.39	Sadness

Ibarretxe-Bilbao et al. (2009) [29]	24	MMSE: 29.8	1.73	14.67	Sadness, fear, anger, disgust, surprise

Kan et al. (2002) [26]	16	MMSE: 26.9	2-3 (range)		Fear, disgust

Lawrence et al. (2007) [22]	17	Nart-IQ: 117.5		22.7	Anger

Narme et al. (2011) [9]	10	MMSE: 28.5 DRS-2: 139.9 FAB: 15.4 BJLOT: 24.3 VOSP: 9.5	2.1		Anger, fear

Sprengelmeyer et al. (2003) [21]	16 (unmedicated)	IQ: 100	1.7	14.6	Fear, sadness, disgust, anger
	20 (medicated)	IQ: 103.2	2.6	30	

Suzuki et al. (2006) [27]	14	MMSE: 28.6	1.6		Disgust

Present study	29 PD	MMSE: 27.6	2.8	26.8	See results
	14 LMDPD	MMSE: 28.1	2.3	24.0	
	15 HMD PD	MMSE: 27.2	3.4	48.7	

H&Y: Hoehn and Yahr's stage (range from I to V) [19]; UPDRS-III: motor section of the Unified Parkinson's Disease Rating Scale [20]; LMD: PD patients with low motor dysfunction; HMD: PD patients with high motor dysfunction; MMSE: Mini Mental State Examination [44]; DRS-2: dementia rating scale-2 (/144) [45]; CDR: clinical dementia rating scale [46]; FAB: frontal assessment battery (/18) [47]; BJLOT: Benton's judgment of line orientation test (/30) [48]; VOSP: visual object and space perception battery [49]; Nart-IQ: IQ estimated using national adult reading test-revised version [50].

**Table 2:** Means (standard deviations) for demographic and clinical characteristics of PD patients and healthy controls in Experiment 1.

Group	Number	Age	BDI-II	MMSE	UPDRS-III	H&Y
PD	29	62.93 (12.78)	12.45 (9.29)	27.62 (1.86)	36.79 (17.29)	2.84 (0.78)
HMD	15	62.53 (13.28)	14.80 (10.17)	27.20 (1.74)	48.73 (14.58)	3.37 (0.69)
LMD	14	63.36 (12.71)	9.93 (7.83)	28.07 (1.94)	24.00 (8.62)	2.29 (0.38)
HC	29	59.07 (10.54)	3.14 (2.61)	28.07 (1.62)		

HMD: PD patients with high motor dysfunction (≧35 on UPDRS-III); LMD: PD patients with low motor dysfunction (<35 on UPDRS-III); HC: healthy controls. BDI-II: Beck Depression Inventory II; MMSE: Mini Mental State Examination; UPDRS-III: motor section of the Unified Parkinson's Disease Rating Scale.

**Table 3:** Means (standard deviations) for demographic and clinical characteristics of PD patients and healthy controls in Experiment 2.

Group	Number	Age	BDI-II	MMSE	UPSRD III
HMD (*n* = 12)	12	65.17 (13.54)	14.25 (9.78)	27.17 (1.90)	50.25 (16.04)
LMD (*n* = 7)	7	67.86 (11.84)	10.29 (3.77)	27.27 (1.80)	21.43 (10.81)
HC (*n* = 15)	5	65.27 (12.19)	7.00 (4.80)	26.93 (3.28)	

HMD: PD patients with high motor dysfunction (≧35 on UPDRS-III); LMD: PD patients with low motor dysfunction (<35 on UPDRS-III); HC: healthy controls. BDI-II: Beck Depression Inventory II; MMSE: Mini Mental State Examination; UPDRS-III: motor section of the Unified Parkinson's Disease Rating Scale.
